# The Characteristics of Moisture and Shrinkage of *Eucalyptus urophylla* × E. Grandis Wood during Conventional Drying

**DOI:** 10.3390/ma15093386

**Published:** 2022-05-09

**Authors:** Lin Yang, Jingting Zheng, Na Huang

**Affiliations:** 1 College of Furnishings and Industrial Design, Nanjing Forestry University, Nanjing 210037, China; jaytine99@njfu.edu.cn (J.Z.); huangnana@njfu.edu.cn (N.H.); 2 Jiangsu Co-Innovation Center of Efficient Processing and Utilization of Forest Resources, Nanjing Forestry University, Nanjing 210037, China

**Keywords:** *Eucalyptus*, moisture content, moisture distribution, moisture flow, shrinkage

## Abstract

High quality lumbers produced from *Eucalyptus* plantations can be used to make higher value-added solid wood products. Moisture flow affects shrinkage, deformation, and quality of *Eucalyptus* wood during conventional drying. In this study, 50 and 100 mm long samples were dried using a conventional drying method. The drying curves, drying rate, moisture content (MC) gradient and distribution, moisture flow, and shrinkage during the drying process were investigated. The results show: Drying was much faster in the first 15 h for all samples and became slow as MC decreased. The drying rate above fiber saturated point (FSP) was about 3.5 times of that below FSP for all samples. The drying rate of 50 mm samples above and below FSP is 1.40 and 1.33 times of 100 mm samples; MC gradients are greater in tangential, radial directions, and cross-sections for both samples when the MC is above FSP, especially at an average MC of 50%. MC gradient along the tangential and radial direction depends on the samples size and MC stages. The short samples have much greater MC gradients than the longer samples above FSP. Moisture distributions on the cross-sections of wood coincide with the moisture gradient in the cross-sections. At an average MC of 50%, the moisture distributions of 50 mm are highly uneven, while they are relatively even in the middle of 100 mm samples, and become much more even at the end of the sample. Moisture distributions become even as MC decreases in all of the samples. Water migration directions vary by state of water. In the short samples, most free water migrates more in the fiber direction from the wood center toward the end surfaces, but bound water diffusion becomes weak. The collapse in the 50 mm samples is significantly larger than that in the 100 mm samples, indicating that the collapse is affected by the dimension of the sample.

## 1. Introduction

Tree species of the *Eucalyptus* genus are widely planted on different continents, due mainly to their fast growth and strong adaptability [1,2,3]. As the predominant type of industrial fast-wood plantations, *Eucalyptus* trees are also planted widely in south China. Until 2015, the *Eucalyptus* plantations occupied an approximate area of 4.5 × 10^6^ hm^2^ [4], which was in response to the economic, social and ecological needs of the country [5].

Most *Eucalyptus* species are generally used to produce low-value products, such as wood-based panels, pulp, laminate and energy products [6]. They are rarely processed into sawn timbers used for higher value-added solid wood products due to the technical processing difficulties associated with high growing tensions, non-ideal fiber orientation, poor dimensional stability, excessive shrinkage, severe collapse and surface checking [7,8,9,10]. However, certain *Eucalyptus* woods have relatively good mechanical strength, few knots, and beautiful wood textures, which have great potential application in high-quality solid wood products, such as furniture, floorings, doors, windows, decorative lines, and other fields [11,12,13,14]. Recently, there has been high interest in increasing the amount of *Eucalyptus* woods as a source of the higher value-added lumbers in Chile, Brazil and China [15,16,17].

The performance of wood products is strongly related to the moisture content, which affects physical, mechanical and chemical properties and the color of wood [18,19,20,21], as well as the processing and use of wood [22,23,24,25]. Thus, wood, especially plantations wood need further treatments to improve wood quality and additional functions [26,27,28,29]. Wood drying can decrease the water in wood to a reasonable level in order to obtain excellent properties, which is paramount for the subsequent processing and service life improvement of wood products [30].

Wood drying shows variations in water migration and its physical state [31]. Water migration is a complex process and involves liquid water and gaseous water movement, depending on the moisture level in wood. Water in wood is divided into free water, or capillary water representing the liquid and gaseous phase above the fiber saturated point (FSP), and bound water representing the gaseous and bound water in the cell wall of the wood, below the FSP [32]. The migration mechanisms of free water and bound water are different. Free water migration is caused by capillary forces, whereas bound water’s migration in the gaseous phase through the cell wall results from diffusion owing to the moisture gradient [33]. Water diffusion in wood is affected by micro-structure, moisture content (MC) and diffusion directions [34,35,36]. Water migration also depends on the principles of water movement. The water migration in the longitudinal direction is higher than that in the radial direction, and the slowest migration is in the tangential direction [37,38]. In hardwoods, the vessels, rays and pits influence the drying characteristics [39,40]. Meanwhile, water migration is also affected by the temperature, relative humidity, the flow of wind, time and wood species [41,42].

There were a lot of studies on the relationship between moisture content and wood’s chemical, physical and mechanical properties [43,44,45,46]. There are some studies [6] on the flow of free water and bound water in *Eucalyptus* wood in different directions and regions during conventional drying. The results provide help in the drying optimization of *Eucalyptus*. In this study, *Eucalyptus urophylla* × E. grandis samples were dried, and the characteristics of moisture distribution, flow of water and shrinkage during conventional drying were investigated and compared to provide technical support for the optimization of conventional drying and parameters to indicate to industries the uses of *Eucalyptus* wood.

## 2. Materials and Methods

### 2.1. Materials

**Figure 1:**
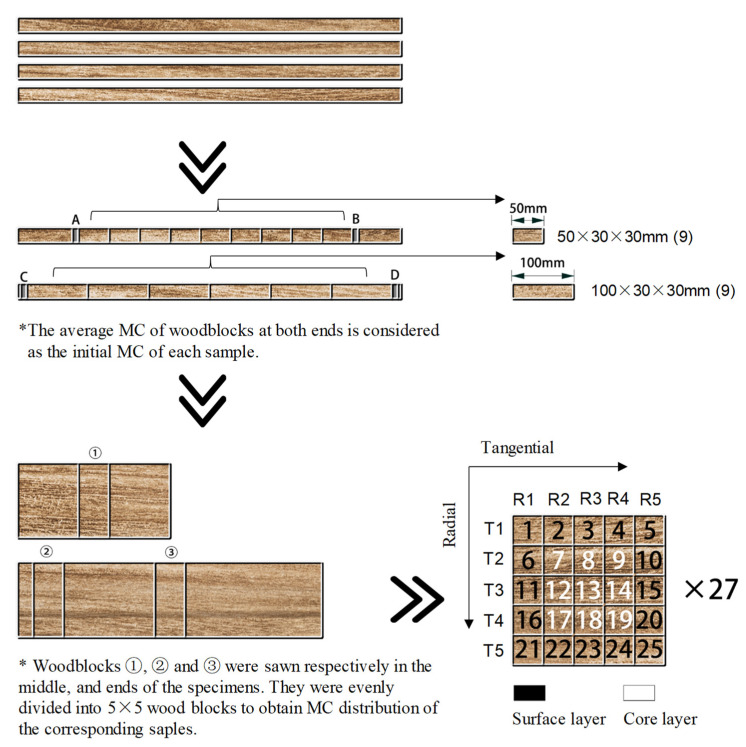
Schematic of the process used to prepare samples.

Six eight-year-old fast-growing *Eucalyptus urophylla* × E. grandis trees were collected from Guangxi Provence, China. The trees were felled and produced into 1000 mm (L) logs and then sealed using plastic films. After that, they were delivered to the lab of Nanjing Forestry University. The logs were processed into boards of 30 (R) × 30 (T) × 1000 (L) mm^3^; after that, they were sawn into two types of end-matched and defect-free samples (nine pieces) with a length of 50 and 100 mm separately, according to the sketch map in Figure 1. The initial MCs were 90% and 98%, respectively.

### 2.2. Equipment and Devices

The drying equipment is an environmental test chamber (DF-408, Nanjing FuDe Instrument Co., Ltd., Nanjing, China) and an electric heat drum wind drying oven (DHG-905, 386-III, Shanghai Cimo Medical Instrument Co., Ltd., Shanghai, China). Other devices are an electronic balance, 0.001 g (Sincere Dedication of Science and Technology Innovation Company, Shanghai, China) and a vernier caliper (CD-20CPX, Mitutoyo, Japan, 0–200 mm/0.01 mm).

### 2.3. Wood Drying

The thin woodblocks of A, B, C and D in Figure 1 were sawn from two ends of 1000 mm boards used to estimate the initial MC of test samples. The MCs were obtained by the oven-dry method which were assumed to be the initial MC of all corresponding samples. According to the initial MCs and weights of samples, the absolute dry weight was obtained. Therefore, the MC of samples during drying was estimated after the samples’ weight was measured. The 50 and 100 mm samples were dried in the environmental test chamber according to the schedule in Table 1. The relative humidity (RH) was constant, while the temperature increased gradually from an ambient temperature of 22 °C to 70 °C with drying time. The samples were taken out from the drying chamber at regular intervals for mass and dimensions’ measurement, and the MCs and shrinkage were calculated. As the MCs of the samples decreased to around 50%, three 50 and 100 mm samples were taken out for mass and dimensions’ measurement, respectively. After measuring, one 8-mm thin wood block was sawn from the middle of each 50 mm sample and two 8-mm thin wood blocks were sawn from one end and the middle of each 100 mm sample, respectively (Figure 1). After that, they were marked and split into 25 small blocks. After weight measuring, they were dried at 103 °C. The MCs of 25 blocks were determined and were used to present the MCs distributions in the samples at an average MC of 50%. The same procedures were conducted as the MCs of the samples decreased to about 30 and 12% MC to investigate the MCs distribution and shrinkage in wood.

### 2.4. Moisture Content Determination

The moisture content of wood in this study was calculated using Equation (1) according to the National Standard of GB/T1931-2009 [47]:

*MC* = (*M*_i_ − *M*_o_)/*M*_o_ × 100% (1)

where *M*_i_ (g) is the initial mass of specimens, *M*_o_ (g) is the mass of oven-dried specimens.

### 2.5. Drying Rate

The drying rate of the 50 mm and 100 mm samples was calculated using Equation (2), to compare the effect of size on the drying rate:

*R* = (*MC*_i_ − *MC*_f_)/*t* × 100% (2)

where *MC*_i_ (%) and *MC*_f_ (%) are the initial and final MC of the samples, respectively, *t* is the time as the MC of the sample decreased from *MC*_i_ to *MC*_f_.

### 2.6. Moisture Contetnt Distribution

As shown in Figure 1, a total of 25 small blocks were prepared, and the MC was measured when the average MC of the samples decreased to about 50, 30 and 12%, respectively. The MCs’ distribution measurement in tangential, radial and cross-section is also shown in Figure 1. For example, T1 indicates the first tangential layer MC of the sample, which is the average MC of one, two, three, four and five wood blocks. T2, T3, T4 and T5 are the second, third, fourth and fifth tangential layer of MC of the sample, respectively. The same measurements are conducted for the MC distribution in the radial direction and cross-section of the samples. The MC distributions along the tangential and radial direction, and the cross-section of the wood corresponding to the three average MCs, were studied and compared.

### 2.7. Shrinkage

Wood normally shrinks as the MC decreases from the FSP, but for wood prone to collapse, it shrinks even when the MC is above the FSP [48]. In this study, the tangential and radial shrinkage of wood during drying is calculated using Equation (3), and the transversal shrinkage is obtained using Equation (4):

*S*_t,r_ = 100 × (*L*_i_ − *L*_f_)/*L*_i_ (3)

*S*_c_ = 100 × (*A*_i_ − *A*_f_)/*A*_i_ (4)

where *L*_i_ and *L*_f_ are the initial and final dimensions in tangential or radial directions of the samples as the MC decreased from *MC*_i_ to *MC*_f_, respectively. *A*_i_ and *A*_f_ are the initial and final areas in cross-sections of the samples corresponding to *MC*_i_ and *MC*_f_, respectively.

## 3. Results and Discussion

### 3.1. Moisture Curves and Drying Rate

**Figure 2:**
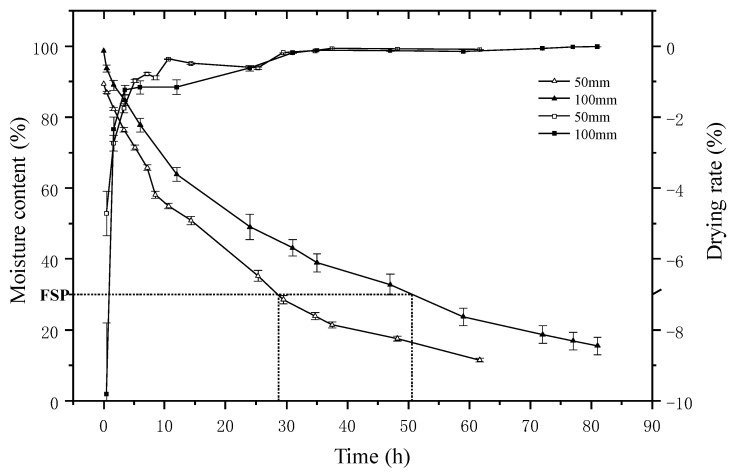
MC and drying rate curves of 50 and 100 mm samples during drying.

The MC and drying rate curves of the 50 and 100 mm samples are presented in Figure 2, and the relevant data are summarized in Table 2. Although the temperature at the beginning is low, at 45 °C, the drying rate is higher for both of the 50 and 100 mm samples. Sharp declines are observed from the MC curves at 10 h and 15 h for 50 and 100 mm samples, respectively. Compared with the 100 mm samples, the drying of the 50 mm samples was faster in this period. After 25 h drying, the temperature increased gradually to 50 °C. However, the drying rates are lower than at the beginning. It took about 30 h and 48 h for the 50 and 100 mm samples, respectively, as the MC decreased to the FSP. The drying rate above the FSP is about 3.5 times of that below the FSP. The drying rate presents a similar tendency to that in a previous study [6], but it is much smaller. It can be clearly seen that drying is much faster as the MC is above the FSP for both samples. Meanwhile, the drying rates of the 50 mm samples above and below the FSP are 1.40 and 1.33 times that of the 100 mm samples, indicating that the drying rate changes with the sample size.

### 3.2. Moisture Content Gradients

#### 3.2.1. Moisture Content Gradient along Tangential and Radial Direction

**Figure 3:**
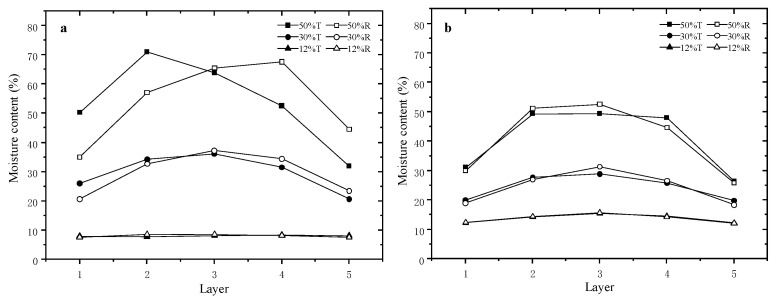
Moisture content gradients along tangential and radial direction. (**a**) 50 mm; and (**b**) 100 mm.

The MC distribution of the 50 and 100 mm samples in the tangential and radial direction at 50, 30 and 12% MC are shown in Figure 3a,b, respectively. The biggest moisture gradients in tangential and radial direction at the average MC of 50% are 39.1% and 32.7% for the 50 mm samples, and 23.0% and 26.7% for the 100 mm samples, respectively. They became 15.4% and 16.5% and 9.1% and 13.0% as the average MC decreased to 30%, respectively, and almost cannot be found as the MC decreased to 12%. These indicate that the moisture gradients are greater above the FSP, especially at an average MC of 50% for both samples. The short samples have a much greater moisture gradient than the longer samples because free water in the surface layer of the wood migrates much faster in short samples. Meanwhile, for the 50 mm samples, the moisture gradient in the tangential direction is greater than that in the radial direction at 50% MC. However, they became almost no different as the MC decreased to 30%. However, for the 100 mm samples, the moisture gradients in the tangential are all smaller than those in the radial direction at both MCs of 50 and 30%. These suggest that more free water migrates in the tangential direction in short samples, but for the 100 mm samples, the migration of the free water becomes stronger in the radial direction. These indicate that the MC gradient along the tangential and radial directions depends on the sample sizes and MC stages.

#### 3.2.2. Moisture Content Gradient in the Cross Section of Wood

**Figure 4:**
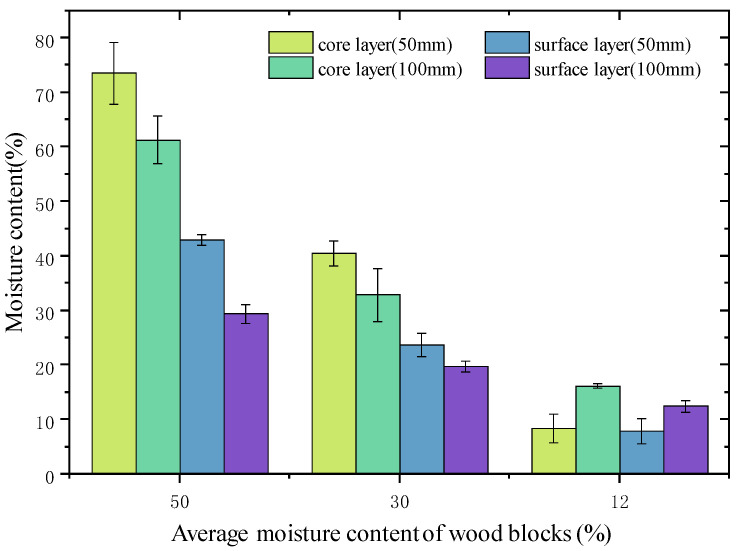
The moisture content gradient between wood surface and core layers.

The MC gradients between surface and core layers in the cross-sections of wood are indicated in Figure 4. The MC gradients between surface and core layers were 30.5% and 31.9% for the 50 and 100 mm samples at an average MC of 50%, respectively, and they became 16.8% and 13.1% at an average MC of 30%, and 0.6% and 3.8% at an average MC of 12%, respectively. These indicate that the MC gradients between the surface and core layers were greater at an average MC of around 50% and became less significant as the average MC decreased for both the 50 and 100 mm samples, showing a similar tendency compared to the tangential and radial directions. At an average MC of 50%, the error bars in the core layers are significantly greater than in the surface layers, indicating the MC differences in the core layers are greater. However, they become smaller in the surface layers showing an even MC. The error bar of the core layers of the 50 mm samples is slightly more extensive than that of the 100 mm samples, indicating a slightly greater MC difference in the core layer of the 50 mm samples. However, for the surface layers, the opposite phenomenon is observed. When the average MC decreased to 30%, the errors in the core and surface layer of the 50 mm samples were almost the same, indicating that the MC is even in the corresponding zones, despite the significant MC gradient between the surface and core layers. However, for the 100 mm samples, the MC differences in the core layers are still greater than that of the 50 mm samples, showing an uneven MC in the core zones. At the final MC stage, the error bars of the surface and core layers for both the 50 and 100 mm samples are almost the same, showing the MC becomes more even in the corresponding zones.

### 3.3. Moisture Distribution in the Cross Sections of Wood

**Figure 5:**
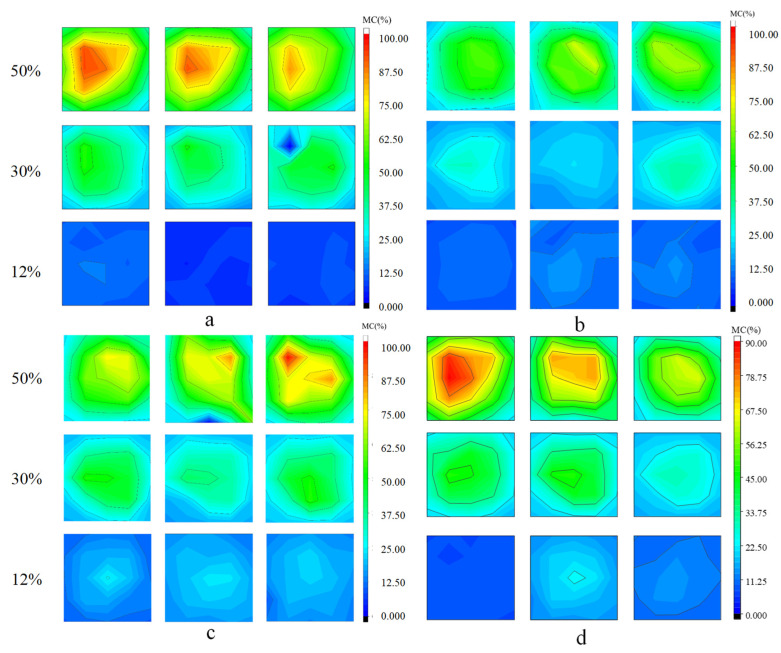
Moisture content distributions in the cross-sections at average MC of 50, 30 and 12%. (**a**) middle sections of each 50 mm sample; (**b**,**c**) end and middle sections of each 100 mm samples, respectively; (**d**) average data of three sections of 50 mm samples (**d left**), middle of 100 mm samples (**d middle**) and end of 100 mm samples (**d right**), respectively.

The MC distribution of every sample and the average data at an average MC of about 50, 30 and 12% are presented in Figure 5 using contour plots. From Figure 5a–c, it can be seen that, although the MC distributions of every sample are not the same as three average MCs, they present as very similar. Therefore, the contour plots of Figure 5d can show the feature of MC distributions of the 50 and 100 mm samples at three average MCs. At an average MC of about 50%, the moisture distributions of the 50 mm samples are hugely uneven, showing a deep red color in the core zones and a green color in the surface zones. Compared with the 50 mm samples, the MC distribution in the middle of the 100 mm samples becomes relatively even and much more even at the end of the sample. When the average MC decreases to 30%, the MC distribution in the 50 mm and in the middle of the 100 mm samples presents as very similar, and become much more even than at the average MC of 50%. Meanwhile, the MC distribution at the end of the 100 mm samples also becomes more even as the MC decreases. When the average MC of the samples decreases to 12%, MC distribution in the 50 mm and the end of 100 mm samples is more even compared with that in the middle of the 100 mm samples, which show a little higher MC in the center of the cross-sections. The MC distribution in the cross-sections indicates a similar tendency to the MC gradient in the cross-sections.

### 3.4. Moisture Flow

Table 3 shows the end and side surface areas, drying rate and the ratios of the total surface areas and drying rates of the 50 to 100 mm samples. The entire surface area of the 50 mm samples is only 57% of 100 mm; however, the total drying rate of the 50 mm samples is 1.24 times of the 100 mm samples. This indicates that, for short samples, most water migrates in the fiber directions from the wood center toward the end surfaces. However, the water migration directions vary by the state of the water. When the MCs are above and below FSP, the ratios of the drying rate of 50 to 100 mm samples are 1.40 and 1.32, respectively. This indicates that, for the 50 mm samples, free water migrates more in the fiber direction above the FSP, but bound water diffusion becomes weak in the fiber direction below FSP. Bound water diffusion in the central parts of the 100 mm samples becomes difficult toward the end surfaces due to the longer transfer path, thus a certain amount of bound water diffuses towards wood side surfaces in the shorter path as the MC is below the FSP.

### 3.5. Shrinkage

**Figure 6:**
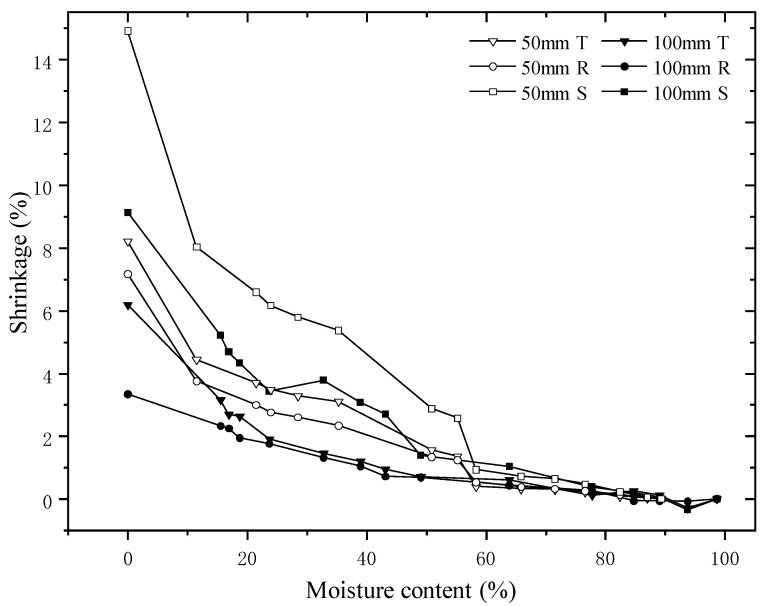
Tangential (T), radial (R) and transversal (S) shrinkage curves of samples during drying.

The tangential, radial and transversal shrinkage of samples during drying are presented in Figure 6. Generally, shrinkages occur in the MC below the saturation of the fiber [49]. However, all samples in our experiments shrank as the MC was above the FSP and increased as the MC decreased. A similar collapse was also observed in a previous study [50]. These shrinkages are termed as collapses resulting from the capillary tension force-induced via free water migration in the lumens of wood cells. As the capillary tension force exceeds the tensile strength limit of the wood’s cell wall, the cell walls deform, and collapse occurs [51,52]. For the collapse of the 100 mm samples, there are no apparent differences in the tangential and radial directions as the MC is above the FSP. The differences become evident when the MC is below the FSP, showing a significant collapse in the tangential direction. However, for the 50 mm samples, the collapse in the tangential direction is greater than when the MC decreases below 60%. The collapse occurred significantly after that MC point and can be observed obviously from the tangential, radial and area curves. The collapse of the 50 mm samples is significantly greater than that of the 100 mm, indicating that the collapse is affected by the dimensions of the samples. For the 50 mm samples, free water migration is much faster than in the 100 mm, resulting in greater capillary tension force which produces more cell collapse.

## 4. Conclusions

*Eucalyptus urophylla* × E. grandis wood was dried using the conventional drying method. The characteristics of the moisture content distribution and the gradient in tangential, radial, and cross-section, moisture flow, and shrinkage were measured and compared during the drying process. The results are summarized: Drying of all samples was much faster in the first 15 h and became slow as the MC decreased, especially when below the fiber saturated point (FSP). The drying rate above the FSP is about 3.5 times of that below the FSP for all samples. The drying rate of the 50 mm samples above and below FSP was 1.40 and 1.33 times of the 100 mm samples; MC gradients are greater in tangential, radial directions and cross sections for both samples when the MC is above the FSP, especially at an average MC of 50%. The MC gradient along the tangential and radial direction is influenced by the samples’ size and MC stages. The MC gradients in short samples are much greater than in the longer samples above the FSP; moisture distributions in cross-sections present a similar tendency to the moisture gradients in the cross-sections. At an average MC of 50%, the moisture distributions of the 50 mm samples are hugely uneven, whereas they are relatively even in the middle of the 100 mm samples and become much more even at the end. Moisture distributions become even with MC decreases for all samples; water migration directions vary by the state of the water. For short samples, free water migrates more in the fiber direction from the wood center toward end surfaces, but bound water diffusion becomes weak in the fiber direction. The collapse of the 50 mm samples is significantly greater than that of 100 mm, indicating the dimension of the sample affects the collapse of wood.

## Figures and Tables

**Table 1:** Drying schedule of the conventional drying.

Time (h)	0	0.3	25	35	48	60	72	>72
Temperature (°C)	22	45	50	55	60	65	70	70
Relative humidity (%)	40	65	65	65	65	65	65	65

**Table 2:** Moisture content, drying time, drying rate of samples during drying.

Samples Type	Initial MC	Final MC	Drying Time (h)			Drying Rate (% h^−1^)		
			>FSP	<FSP	Total	>FSP	<FSP	Total
50 mm	89.3	11.5	29.5	32.2	61.7	−2.01	−0.57	−1.26
100 mm	98.7	15.5	47.5	34.0	81.5	−1.44	−0.43	−1.02

**Table 3:** Surface areas and drying rate of samples.

Sample Type	Surface Area (cm^2^)			Ratio of Surface Area	Ratio of Drying Rate		
	End	Side	Total	Total	>FSP	<FSP	Total
50 mm	1800	6000	7800	0.57	1.40	1.32	1.24
100 mm	1800	12,000	13,800				

## Data Availability

Not applicable.

## References

[1] Morales P., Gentina J.C., Aroca G., Mussatto S.I. (2017). Development of an acetic acid tolerant *Spathaspora passalidarum* strain through evolutionary engineering with resistance to inhibitors compounds of autohydrolysate of *Eucalyptus globulus*. Ind. Crops Prod..

[2] Carignato A., Moraes C.B.d., Zimback L., Mori E.S. (2018). Genetic Resistance to Rust of *Eucalyptus urophylla* Progenies. Floresta E Ambiente.

[3] Wessels C.B., Nocetti M., Brunetti M., Crafford P.L., Proller M., Dugmore M.K., Pagel C., Lenner R., Naghizadeh Z. (2020). Green-glued engineered products from fast growing *Eucalyptus* trees: A review. Eur. J. Wood Wood Prod..

[4] Williams R.A. (2015). Mitigating biodiversity concerns in *Eucalyptus* plantations located in South China. J. Biosci. Med..

[5] Tan X., Yu X.W., Zhang X., Li F., Liu Y., Ouyang X., Wen Y.Z. (2020). Propagation characteristics of LoRa signal at 433 MHz channel in eucalyptus plantation environment. J. For. Eng..

[6] Monteiro T.C., Lima J.T., Hein P.R.G., da Silva J.R.M., Neto R.d.A., Rossi L. (2021). Drying kinetics in *Eucalyptus urophylla* wood: Analysis of anisotropy and region of the stem. Dry. Technol..

[7] Yang J.L., Waugh G. (2001). Growth stress, its measurement and effects. Aust. For..

[8] Crafford P., Wessels C. (2016). A potential new product for roof truss manufacturing: Young, green finger-jointed *Eucalyptus grandis* lumber. South J. Sci..

[9] Ananias R.A., Sepulveda-Villarroel V., Perez-Pefia N., Torres-Mella J., Salvo-Sepulveda L., Castillo-Ulloa D., Salinas-Lira C. (2020). Radio Frequency Vacuum Drying of *Eucalyptus* nitens Juvenile Wood. Bioresources.

[10] Franca F.J.N., Maciel A.P.V., Franca T.S.F.A., Silva J.G.M., Batista D.C. (2019). Air-drying of Seven Clones of *Eucalyptus grandis* × *Eucalyptus urophylla* Wood. Bioresources.

[11] Liu H., Zhang Y., Wu Z. (2018). Effects of Ultrasound Pretreatment on Microstructure and Drying Characteristics of *Eucalyptus urophylla* x E. grandis. Bioresources.

[12] Chen Y., Zhu J. (2019). Study on bending characteristics of fast growing eucalyptus bookcase shelves by using burgers model. Wood Res..

[13] Zhao X.Y., Huang Y.J., Fu H.Y., Wang Y.L., Wang Z., Sayed U. (2021). Deflection test and modal analysis of lightweight timber floors. J. Bioresour. Bioprod..

[14] Zhang L., Chen Z.H., Dong H.R., Fu S., Ma L., Yang X.J. (2021). Wood Plastic Composites Based Wood Wall’s Structure and Thermal Insulation Performance. J. Bioresour. Bioprod..

[15] Teixeira T.d.O.B., Silva M.L.d., Jacovine L.A.G., Valverde S.R., Silva J.d.C., Pires V.A.V. (2009). A percepção sobre o uso da madeira de eucalipto pelos fabricantes do polo moveleiro de Ubá-MG. Rev. Árvore.

[16] Ananias R.A. Drying developments on Drying of Chilean hardwoods. Academy Lecture Prepared for the IAWS Annual Meeting. Proceedings of the Biosustainable Materials: Key to a Better Future.

[17] Zhen X., Deng Y., Li Y.Z., Zhu X.M. (2021). Design and experiment of pruning machine for eucalyptus trees. J. For. Eng..

[18] Kask R., Lille H., Kiviste M., Kruus S., Lääne J.O. (2021). Effect of Soaking/Oven- Drying on Mechanical and Physical Properties of Birch (*Betula* spp.). Plywood. Drv. Ind..

[19] Herrera-Díaz R., Sepúlveda-Villarroel V., Pérez-Peña N., Salvo-Sepúlveda L., Salinas-Lira C., Llano-Ponte R., Ananías R.A. (2018). Effect of wood drying and heat modification on some physical and mechanical properties of radiata pine. Dry. Technol..

[20] Shen Y., Gao Z., Hou X., Chen Z., Jiang J., Sun J. (2020). Spectral and thermal analysis of *Eucalyptus* wood drying at different temperature and methods. Dry. Technol..

[21] Klement I., Vilkovská T., Baranski J., Konopka A. (2019). The impact of drying and steaming processes on surface color changes of tension and normal beech wood. Dry. Technol..

[22] Passarini L., Hernández R.E. (2016). Effect of the desorption rate on the dimensional changes of *Eucalyptus* saligna wood. Wood Sci. Technol..

[23] Liu H., Zhang J., Jiang W., Cai Y. (2019). Characteristics of Commercial-scale Radio-frequency/Vacuum (RF/V) Drying for Hardwood Lumber. Bioresources.

[24] Yin Q., Liu H.-H. (2021). Drying stress and strain of wood: A Review. Appl. Sci..

[25] Zhou T., Liu H. (2022). Research Progress of Wood Cell Wall Modification and Functional Improvement: A Review. Materials.

[26] Feng X.H., Chen J.Y., Yu S.X., Wu Z.H., Huang Q.T. (2022). Mild hydrothermal modification of beech wood (Zelkova schneideriana Hand-Mzt): Its physical, structural, and mechanical properties. Eur. J. Wood Wood Prod..

[27] Yang Y.Q., Xu W., Liu X., Wang X.D. (2021). Study on Permeability of Cunninghamia Lanceolata Based on Steam Treatment and Freeze Treatment. Wood Res..

[28] Fang L., Zeng J., Zhang X., Wang D. (2021). Effect of Veneer Initial Moisture Content on the Performance of Polyethylene Film Reinforced Decorative Veneer. Forests.

[29] Nishiyama Y.H. (2021). Retrieving Structural Information from Scattering and Attenuation Data of Transparent Wood and (Nano)paper. J. Bioresour. Bioprod..

[30] Zen L., Magalhães T., Henrique J., Klitzke R. (2020). Drying methods to evaluate the quality of *Eucalyptus* sawn timber. Aust. J. Basic Appl. Sci.

[31] Monteiro T.C., Lima J.T., Silva J.R.M.d., Rezende R.N., Klitzke R.J. (2020). Water flow in different directions in Corymbia citriodora wood. Maderas. Cienc. Tecnol..

[32] Siau J.F. (1984). Transport Processes in Wood.

[33] Skaar C. (1972). Water in Wood.

[34] Cao M.D., Zhang X.X., Ren W.T., Zhu J.W., Wang H.K., Xu H.C., Yu Y. (2021). Effect of drying methods on the cell wall pore structure of Phyllostachys edulis. J. For. Eng..

[35] Monteiro T.C., Lima J.T., Abreu Neto R., Ferreira C.A. (2021). Importance of Pits in Corymbia Citriodora (Hook.) K.D. Hill & L.A.S. Johnson (Myrtaceae) Wood Permeability. Floresta E Ambiente.

[36] Liu J.Y., Simpson W.T. (1999). Two-stage moisture diffusion in wood with constant transport coefficients. Dry. Technol..

[37] Engelund E.T., Thygesen L.G., Svensson S., Hill C.A. (2013). A critical discussion of the physics of wood–water interactions. Wood Sci. Technol..

[38] Monteiro T.C., Lima J.T. (2020). Water Flow Through the Pits in *Eucalyptus urophylla* Wood. Floresta E Ambiente.

[39] Barauna E.E.P., Lima J.T., Vieira R.d.S., da Silva J.R.M., Monteiro T.C. (2014). Effect of Anatomical and Chemical Structure in the Permeability of “AMAPA” wood. Cerne.

[40] Monteiro T.C., Lima J.T., Hein P.R.G., da Silva J.R.M., Trugilho P.F., Andrade H.B. (2017). Efeito DosElementos Anat^omicos Da Madeira Na Secagem DasToras de *Eucalyptus* e Corymbia. Sci. For..

[41] Taghiyari H.R., Habibzade S., Tari S.M.M. (2014). Effects of Wood Drying Schedules on Fluid Flow in Paulownia Wood. Dry. Technol..

[42] Sik H.S., Choo K.T., Zakaria S., Ahmad S., How S.S., Chia C.H., Yusoff M. (2010). Dimensional Stability of High Temperature-Dried Rubberwood Solid Lumber at Two Equilibrium Moisture Content Conditions. Dry. Technol..

[43] Gao Y.Q., Li Y.Y., Ren R.Q., Chen Y., Gao J.M. (2021). Effect of weak acid modification on the structure and properties of heat-treated Chinese fir. J. For. Eng..

[44] Pelit H., Emiroglu F. (2020). Effect of Water Repellents on Hygroscopicity and Dimensional Stability of Densified Fir and Aspen Woods. Drv. Ind..

[45] Sedlar T., Šefc B., Drvodelić D., Jambreković B., Kučinić M., Ištok I. (2020). Physical Properties of Juvenile Wood of Two Paulownia Hybrids. Drv. Ind..

[46] Tu D.Y., Chen C.F., Zhou Q.F., Ou R.X., Wang X.J. (2021). Research progress of thermo-mechanical compression techniques for wood products. J. For. Eng..

[47] (2009). Method for Determination fo the Moisture Content of Wood.

[48] Hernández R.E., Pontin M. (2007). Shrinkage of Three Tropical Hardwoods Below and Above the Fiber Saturation Point. Wood Fiber Sci..

[49] Li Z., Jiang J.L., Lyu J.X., Cao J.Z. (2021). Orthotropic Viscoelastic Properties of Chinese Fir Wood Saturated with Water in Frozen and Non-frozen States. For. Prod. J..

[50] Yang L., Liu H.H. (2021). Study of the collapse and recovery of *Eucalyptus urophydis* during conventional kiln drying. Eur. J. Wood Wood Prod..

[51] Gonya N.A.S., Naghizadeh Z., Wessels C.B. (2022). An investigation into collapse and shrinkage behaviour of *Eucalyptus grandis* and E*ucalyptus grandis-urophylla* wood. Eur. J. Wood Wood Prod..

[52] Liu H., Gao J., Chen Y. (2015). Effects of Pre-Freezing Prior to Drying upon Some Physical and Mechanical Properties of *Eucalyptus urophylla* × *Eucalyptus grandis* Wood. Bioresources.

